# Review of an intelligent indoor environment monitoring and management system for COVID-19 risk mitigation

**DOI:** 10.3389/fpubh.2022.1022055

**Published:** 2023-01-10

**Authors:** Rita Wiryasaputra, Chin-Yin Huang, Endah Kristiani, Po-Yu Liu, Ting-Kuang Yeh, Chao-Tung Yang

**Affiliations:** ^1^Department of Industrial Engineering and Enterprise Information, Tunghai University, Taichung, Taiwan; ^2^Department of Informatics, Krida Wacana Christian University, Jakarta, Indonesia; ^3^Department of Computer Science, Tunghai University, Taichung, Taiwan; ^4^Division of Infection, Department of Internal Medicine, Taichung Veterans General Hospital, Taichung, Taiwan; ^5^Ph.D. Program in Translational Medicine, National Chung Hsing University, Taichung, Taiwan; ^6^Rong Hsing Research Center for Translational Medicine, National Chung Hsing University, Taichung, Taiwan; ^7^Department of Post-Baccalaureate Medicine, College of Medicine, National Chung Hsing University, Taichung, Taiwan; ^8^Genomic Center for Infectious Diseases, Taichung Veterans General Hospital, Taichung, Taiwan; ^9^Research Center for Smart Sustainable Circular Economy, Tunghai University, Taichung, Taiwan

**Keywords:** building ventilation, COVID-19, indoor environmental quality, intelligent monitoring, SARS-CoV-2

## Abstract

The coronavirus disease (COVID-19) outbreak has turned the world upside down bringing about a massive impact on society due to enforced measures such as the curtailment of personal travel and limitations on economic activities. The global pandemic resulted in numerous people spending their time at home, working, and learning from home hence exposing them to air contaminants of outdoor and indoor origins. COVID-19 is caused by the Severe Acute Respiratory Syndrome Coronavirus 2 (SARS-CoV-2), which spreads by airborne transmission. The viruses found indoors are linked to the building's ventilation system quality. The ventilation flow in an indoor environment controls the movement and advection of any aerosols, pollutants, and Carbon Dioxide (CO_2_) created by indoor sources/occupants; the quantity of CO_2_ can be measured by sensors. Indoor CO_2_ monitoring is a technique used to track a person's COVID-19 risk, but high or low CO_2_ levels do not necessarily mean that the COVID-19 virus is present in the air. CO2 monitors, in short, can help inform an individual whether they are breathing in clean air. In terms of COVID-19 risk mitigation strategies, intelligent indoor monitoring systems use various sensors that are available in the marketplace. This work presents a review of scientific articles that influence intelligent monitoring development and indoor environmental quality management system. The paper underlines that the non-dispersive infrared (NDIR) sensor and ESP8266 microcontroller support the development of low-cost indoor air monitoring at learning facilities.

## 1. Introduction

The COVID-19 outbreak has had a significant impact on daily lives affecting work, school, the healthcare system, and the pursuit of pleasure. Since the outbreak became an altering global health crisis, the pandemic is nearing its end. Nevertheless, the public faces the threat of COVID-19 common symptoms such as coughs, headaches, eye irritation, dizziness, fatigue, and death. The rapid transmission of the deadly virus led to a strong emphasis on health precautions and prevention techniques by government officials and health institutions worldwide to curb its spread. Several situations can exacerbate the number of COVID-19 cases, particularly from a socio-demographic level, including lack of essential services, slums and impoverished settlements, the density of public transportation, the duration of a journey, and the state of a country's infrastructure. Furthermore, climatic factors such as temperature, airflow, air quality, and humidity may also contribute to increased COVID-19 cases. In Spain's capital city of Madrid, the mortality rate was 312 deaths per 100,000 inhabitants on 5 May 2020 (1). Individuals suffering from respiratory disease and people aged 60 years and above who were exposed to poor air quality (2) had a higher mortality rate. During Spain's first outbreak, the lifestyle of its residents underwent a drastic change because the entire Spanish population was placed under a 24/7 lockdown for 45 days. As such, people were mandated to stay at home and could only leave to perform essential activities (1). In the education sector, the pandemic affected class attendance, academic performance, and children's health. Consequentially, numerous countries had to grapple with a health crisis as well as economic shocks that affected human capital, productivity, and society.

According to the World Health Organization, the primary mechanism of COVID-19 transmission is between people, particularly in situations where there is extended and unprotected virus exposure (3). The SARS-CoV-2 virus—which causes COVID-19—is emitted from a contagious person's respiratory system and transmitted to a responsive host. There are three major routes for the spread of COVID-19, namely droplet transmission, close contact, and airborne transmission (4–8). Particulate matter (PM) is also known as particle pollution in the air that comes in solid and/or liquid form. The size of the virus particles ranges from sub-micrometers to a few micrometers whilst the pathogens are contained in fluid-based particles that are aerosolized from respiratory tract sites during activities such as coughing, breathing, sneezing, and speaking (9). Previous studies found that indoor air circulation is a crucial factor behind virus viability, therefore, poor ventilation, loud vocalization, density occupancy, and stay duration (7, 10, 11) increase environmental risk factors in transmitting the virus. The research conducted by Baboli revealed that the essential condition which harbors the presence of the virus indoors includes low levels of humidity and temperature, a high PM level, and the absence of any air filtration (12).

In addition, outdoor temperatures are hotter than usual due to global warming which can influence people's choices to spend time indoors working, learning, and relaxing. Therefore, it is important to measure indoor air quality as it is one of the most significant real-time indicators for today's urban environment given how it significantly influences human health, safety, and comfort (13); the Air Quality Index (AQI) represents a quantitative level of air pollution. Indoor air pollution is influenced by how building ventilation systems are planned, managed, and maintained thus when rooms are not well-ventilated, there is a risk of cross-infection. Minimizing indoor air pollution is the first and best measure to ensure indoor environmental quality whereas providing sufficient ventilation supports mitigation strategies of airborne disease transmissions. Adequate ventilation helps to control the movement and advection of any aerosols, pollutants, and CO_2_ created by indoor sources/occupants (14). One of the essential parameters of indoor CO_2_ level scales quite accurately with the number of individuals in a room and their activity level (8, 15). As such, measuring CO_2_ levels can indicate the presence of sufficient ventilation which in turn, aids in the prevention of respiratory virus infections in humans. Indoor CO_2_ monitoring is, thus, an effective technique to assess an individual's COVID-19 risk. However, high, or low CO_2_ levels cannot directly detect COVID-19 whilst close contact poses the highest risk for COVID-19 transmission.

The SARS-CoV-2 virus will not disappear given that it has anchored itself as part of our lives. Even though people have received COVID-19 vaccinations and normal activities resumed by wearing face masks, the risk of reinfection still lingers. The SARS-CoV-2 virus infects the upper respiratory system and is dangerous and potentially lethal given that infected persons could develop severe symptoms and illnesses while those who have recovered may suffer from “Long COVID”. A COVID-19 infection could lead to hospitalizations which can be particularly expensive in some countries. The elderly, unvaccinated people and persons with underlying medical conditions are the most at-risk groups due to a weakened immune system. The SARS-CoV-2 virus is known for its rapid transmission, yet no person is naturally immune to the virus underscoring the extreme danger of the illness. Essentially, maintaining a safe and healthy environment is crucial to breaking the COVID-19 chain of infection. In terms of COVID-19 risk mitigation, zoning can be implemented by remote sensing and Geographic Information System (GIS) with several parameters such as hazard data, socioeconomic data, and biophysical data enabled to categorized of five zones that are representative of colors, beginning with a very high-risk zone (red), the high-risk (orange), the moderate zone (blue), the low-risk zone (green), until the very low zone (pink) (16–18). Certain countries such as Germany, India, the USA, and South Africa have experienced more than two COVID-19 waves (19). Meanwhile, other countries have reported mutations of the SARS-CoV-2 virus in addition to cases of the monkeypox disease that also spreads through respiratory droplets (20). Most disease transmissions occur indoors thus the development of intelligent air monitoring and management system—boosted by cutting-edge technologies, like cloud computing and the Internet of Things (IoT)—is vital. Intelligent indoor air monitoring comprises many sensors that are sold at various prices in the marketplace. Low-cost sensors based on Electrochemical Cell (EC), Metal-oxide Semiconductor (MOS), Non-Dispersive Infrared (NDIR), nephelometry, and Optical Particle Counters (OPC) promise the indoor air-monitoring as user-friendly, easy deployment portable devices (15, 21, 22). This study aims to analyze the potential of indoor air monitoring and alternative solutions to minimize disease transmissions. In this paper, the self-assembly of an indoor monitoring system using inexpensive sensors is aimed to support the realization of a healthy indoor environment, particularly in reducing COVID-19 risks.

The structure of this paper is as follows: the first section reviews the background and previous research, the method is explained in methods and result section presents the research results. To deepen the understanding of other objective studies, features a discussion section. The conclusion is outlined in conclusion section along with future research directions.

## 2. Methods

To accomplish this study, several electronic databases inclusive of Scopus, Web of Science (WoS), ScienceDirect, IEEE Xplore, Google Scholar, and EBSCO databases as well as the keywords “indoor ventilation transmission COVID19”, “indoor room monitoring COVID19”, “air quality index COVID19”, “cloud computing COVID19”, “internet of things COVID19” were used in systematic searching. The Preferred Reporting Items for Systematic Reviews and Meta-Analyses (PRISMA) approach is an evidence-based minimum set of items to conduct a Systematic Review for the identification, screening, evaluation, and analysis of the eligibility of all published studies relevant to a certain Research Topic (23).

**Figure 1:**
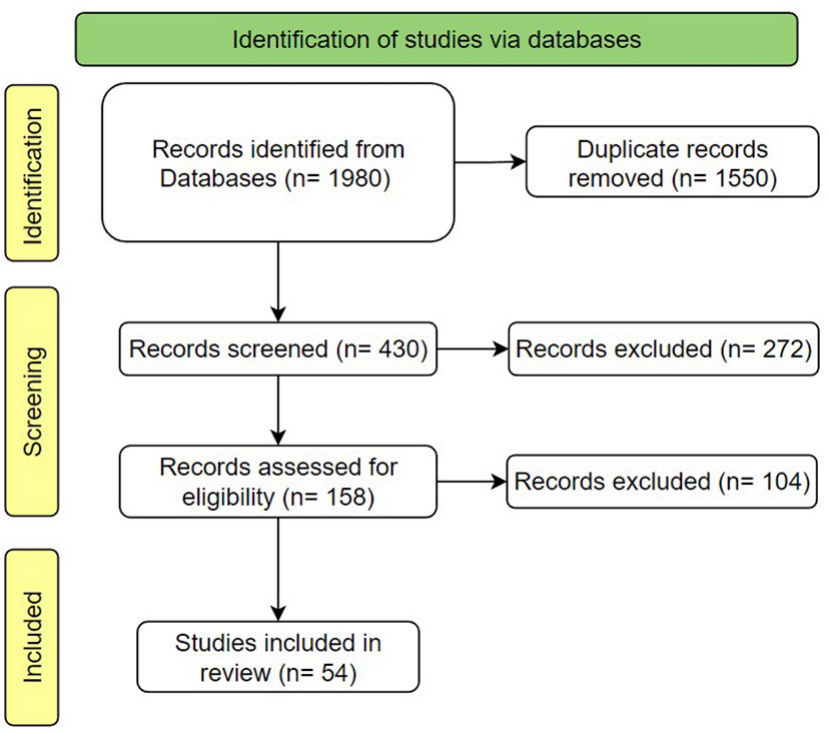
PRISMA flow diagram.

There were 1,980 extracted papers from the electronic databases mentioned above which have been peer-reviewed and were published between 2019 and 28th February 2022. After the extraction process, 1,550 duplicate records were removed, and 430 papers were screened based on the title and the abstract. In total, there were 158 eligible articles, but 104 were excluded for a few reasons: not available in English, it was a conference abstract, or the focus was not on indoor monitoring. A file (“.ris” format) in the Mendeley Reference Manager contained 54 full-text electronic English references. Figure 1 shows the working of PRISMA method in the flow diagram.

## 3. Results

**Figure 2:**
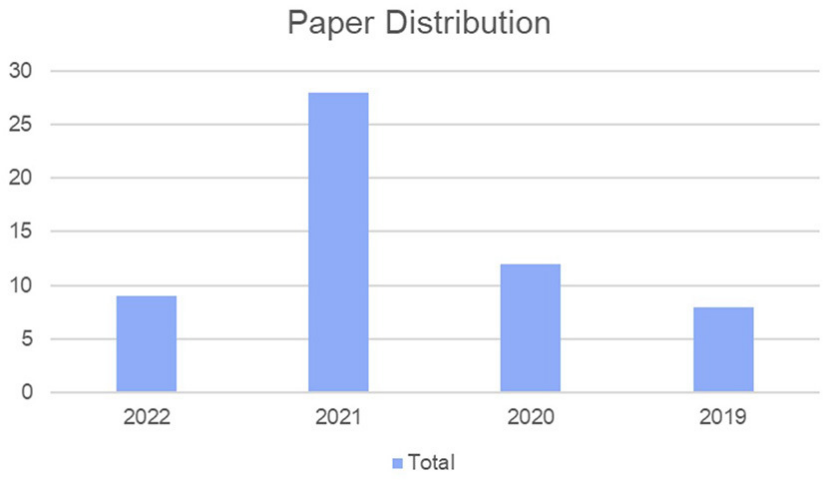
Paper distribution.

The corpus contained five databases in which Google Scholar had the highest percentage and the WoS had the lowest, as shown in Table 1. Figure 2 shows the distribution of the examined publications which has increased since 2019 and reached a peak in publications in 2021.

**Table 1:** Percentage papers from database.

**Database**	**Percentage (%)**
Scopus	4.4
Science direct	26.7
Google scholar	34.5
Web of science (WoS)	0.4
IEEE Xplore	1.4
EBSCO	32.6

The journals from Elsevier had the most publications and the most frequently identified was the journal Sensors from MDPI publishers. Table 2 shows the classification of study types conducted by the researchers which consist of 45 experimental study papers and 9 review papers from various interdisciplinary studies.

**Table 2:** Classification of study type.

**Study**	**Type of study**
Adam (5), Agarwal (2), Burridge (4), Chojer (15, 22), Dinoi (24), Elsaid (25), Eykelbosh (8), Hajjaji (23), Noorimotlagh (3), Singh (26)	Review
Aguilar (27), Assante (28), Asthana (29), Baboli (12), Barrio (30), Bidila (31), Carlotti (32), Chandel (16), Chen (33), Chen (34), Choe (35), Cicceri (36), Dhanalakshmi (37), Dinh (38), Dominguez (1), Gilio (39), Goyal (40), Guo (41), Hoang (42), Hou (7), Huang (43), Jo (44), Kaliszewski (45), Kanga (17, 18), Kenarkhoohi (46), Khan (47), Kim (48), Ko (49), Ladekar (50), Li (51), Marques (52), Miller (10), Ng (53), Palanisamy (54), Pareek (55), Peng (56), Petrovic (57), Pietrogrande (58), Rantas (59), Ren (6), Rivas (60), Stufano (61), Vanus (62), Wall (63), Wang (64), Yang (65), Yang (66)	Experimental study

Table 3 represents the classification data references present in the corpus. The Building and Environment journal in the building domain focuses on building science, urban physics, and human interaction with the indoor and outdoor built environment (67). The Sensors journal accommodates the original contribution submission in science and technology of sensors and their application (68). The Journal of Sustainable Cities and Society specializes in environmentally sustainable and socially resilient cities (69). The Environmental Research journal welcomes a multi-disciplinary approach aimed at anthropogenic issues of global relevance and applicability in a wide range of environmental disciplines and demonstrates environmental application in the real-world context (70).

**Table 3:** Classification data references present in the corpus.

**Journal**		**Domain**	
Elsevier	29	Building	8
		Sustainable Cities and Society	3
		Environment	6
		Others	12
MDPI	6	Sensors	4
		Others	2
Others	19	Others	19

Chinese academics and researchers have contributed toward COVID-19 mitigation, meanwhile, academics and researchers from Italy and the Republic of Korea have demonstrated a strong interest in deepening the study concerning COVID-19 indoor monitoring topics. Table 4 represents the classification data based on the Author's country. The experimental study on indoor monitoring used a wide range of locations comprising university classrooms, university laboratories, university offices, classrooms in daycare centers, hospital rooms, and houses.

**Table 4:** Author's country.

**Author's country**	**#Papers**
Egypt	1
Canada	1
China	7
France	1
India	6
Iran	3
Italy	7
Poland	1
Portugal	1
Republic of Korea	6
Romania	1
Spain	4
Singapore	2
USA	4
Tunisia	1
UK	1
Taiwan	3

# means numbers of paper.

Researchers expanded their focus beyond measuring CO_2_ by also measuring temperature and humidity (27, 35, 65, 66). To simplify the monitoring process, wireless technologies such as ZigBee were additionally utilized (52, 65, 71). Although researchers deployed their low-cost indoor monitoring system, no explanation has been provided about the significance of monitoring measures in reducing COVID-19 cases. Regardless, indoor monitoring data can be used to predict COVID-19 risks.

Table 5 shows the potential area of indoor air monitoring, the duration of observation, and the sensors/technologies used in building non-commercial indoor air monitoring monitors. Kim and the team evaluated indoor air quality based on the national guidance centers in South Korea and discovered that most indoor air contaminants were found during the opening hours of daycare centers (a public area) even though the centers operated per the Regulations for Building Systems (Ministry of Land, Infrastructure, and Transport) and the Indoor Air Quality Control Act (Ministry of Environment) in South Korea (72). They found that CO_2_, PM_10_, and PM_2.5_ possessed the capability of becoming a media for infectious viruses and that the density and activity of indoor occupants are the driving factors of indoor air contaminants.

**Table 5:** Indoor air monitoring sensors/technology.

**Study**	**Case/ duration**	**Observation time**	**The main objective**	**Keyword**	**Technology/ sensors**
Aguilar (27)	Educational building/2 h	n.a	Assement ventilation rate and ventilation strategies in educational building	Indoor air quality, coronavirus, COVID-19, lockdown, social distancing, air purifiers, Air Quality Index, AQI forecasting, Indoor air quality improvement, impact or effectiveness, ventilation, face masks	HOBO MX1102
Barrio (30)	Classrooms/6 h	3rd−13th March 2020, 11th−22nd January 2021	Analyzing energy efficiency and indoor environment, compare indoor environmental parameters	Monitoring, COVID, IAQ, IEQ, natural ventilation, HRV, temperature, CO_2_	n.a
Bidila (31)	Classrooms/ more than an hour	6 months	Measuring the density of people with the level of TVOC in a room	Indoor air quality, sensor systems, Internet of Things, risk of COVID-19 transmission	ESP8266 microcontroller, HTU21D, CCS811, MQTT protocol
Carlotti (32)	Rooms/n.a	n.a	Determining the airborne transmission of COVID19 through droplets in suspension	COVID-19, aeraulics, Wells model, Doseeffect, VLES, indoor air quaility, classroom	Software FDS Classroom simulation by NIST
Choe (35)	Classrooms/n.a	n.a	Evaluating the improvement of IAQ, identification the characteristics and factors affecting IAQ	School, Particulate matter, carbon dioxide, indoor air quality, perceived air quality, air purifier	PPD42NS, S-300-3V sensor CO2
Dinh (38)	n.a	n.a	Developing a non-dispersive infrared (NDIR) analyzer with a wide range of measurements (ppmv to percentage levels) for measuring carbon dioxide (CO2) in an indoor environment	carbon dioxide; indoor air; NDIR; pathlength; interference	Non-dispersive infrared (NDIR) analyzer
Gilio (39)	School classrooms/5 h	18th January−8th February 2021	Developing a surveillance activity based on real-time monitoring of CO_2_ levels as a proxy of SARSCoV-2 transmission risk	CO_2_, COVID-19, SARS-CoV-2 transmission risk mitigation, Indoor ventilation conditions, School re-opening, NDIR sensor	commercial NoseC non-dispersive infrared (NDIR) sensors
Ladekar (50)	House or an industry at various places/n.a	n.a	Notification by email and SMS	indoor air quality, real time air quality monitoring, Internet of Things, visualization, alert	NodeMCU ESP8266, Raspberry pi, AWS IoT (MQTT), Kibana visualization, GP2Y20100UF, MH-Z19 NDIR, Gas grove
Ng (53)	Dry lab/n.a	n.a	Deploy the ScAlN-based pyroelectric detectors utilizing 8-inch wafer level technology and 12 percent Scdoped AlN deposited at 200°C	Pyroelectric detector, Scandium aluminum nitride (ScAlN), CO_2_ gas sensor, MEMS, CMOS compatible, Non-dispersive infrared	NDIR CO_2_ gas sensing
Vanus (62)	training center room/n.a	Spring and fall 2018	Investigate the possibility of improving the accuracy of CO_2_ forecasts in Smart Home Care (SHC) by determining the occupancy hours of a monitored SHC room using IBM SPSS software tools in the IoT	Smart Home Care (SHC), monitoring, prediction, trend detection, Artificial Neural Network (ANN), Radial Basis Function (RBF), Wavelet Transformation (WT), SPSS (Statistical Package for the Social Sciences) IBM, IoT (Internet of Things), Activities of Daily Living (ADL)	Siemens QPA2062
Wall (63)	kitchen/n.a	28th July−11th August 2020, 5th−19th November 2020	Developing IAQ monitoring based on four layer IoT architecture	IoT, Indoor, air, quality, analytics, tutorial	ESP32 microcontroller, Bosch BME680 sensor
Kim (48)	Daycare center/12 h	1 year (Opening 12 h) September 2019–June 2020	Monitoring and evaluating indoor air contaminant variables quality based on the national guidance centers in Korea	Daycare center, air contaminant, indoor air quality, field study, COVID-19, machine learning	CESCO EM2001
Marques (52)	Laboratory environment/n.a	2 months	Building the architecture of low-cost and open-source CO2 real-time monitoring (iAirCO2) based on IoT system	AAL (Ambient Assisted Living), enhanced living environments, Health informatics, IAQ (Indoor Air Quality), IoT (Internet of Things), Smart cities	MH-Z19 NDIR (CO2) produced Winsensor, ESP8266 Thing Dev (Sparkfun) microcontroller
Salman (71)	Office/max 30 min	n.a	Building an IAQ map of building with a low-cost Wireless Sensor Network (WSN), streaming IAQ data for real time processing and analysis	wireless sensor network, spatial prediction, indoor air quality	Sensirion SCD 30 (NDIR), XBee communication, ARM microcontroller
Yang (65)	Campus classroom/15 h	During student final exam week	Developing a prototype of an intelligent indoor environment monitoring system using sensors in the Cloud computing and Big Data environment based on an ideal Temperature and Humidity Index (THI) value	Sustainability, iDEMS, environmental sensors, cloud computing, big data, Hbase	ZigBee WSN, temperature sensor, humidity sensor, Carbon Monoxide (CO), Formaldehyde, Volatile Organic Compounds (VOCs), HBase, CO_2_ sensor, OpenStack
Yang (66)	library/n.a	n.a	Evaluating current PM monitoring methods, Presenting the incorporation of AI and IoT	Particulate Matter (PM), AIoT, Microsensor, PM monitoring, IoT	NBIoT, temperature sensor, humidity sensor, Formaldehyde, Volatile Organic Compounds (VOCs), PM_2.5_, CO_2_ sensor

Most of the research found a correlation between seasonality and increased COVID-19 cases. The researchers from our study concluded that SARS-CoV-2 spreads slower in the summer than in the winter demonstrating the significant role of humidity and temperature. Elsaid (25) provided guidelines and recommendations to achieve indoor air quality in air-conditioned areas. It includes ensuring the height of an exhaust air line is at least 5 meters from the end of a building's ceiling and increasing the exhaust fan size. Another suggestion is to reach an indoor temperature of 25–27°C and relative humidity of 50–70% which can suppress the SARS-CoV-2 virus from spreading (25, 73). However, Meraj and his teams (74) found that the spreading of COVID-19 was not influenced by temperature significantly when they investigated three areas with distinct climatic (subtropical, desertic, coastal) in India.

Agarwal (2) found that there was a positive correlation between air pollution (NO_2_ and PM_2.5_) and COVID-19 contamination in the air quality index. Natural ventilation uses vents, louvers, windows, or mechanical ventilation systems. In addition, an effective non-medical action that can considerably reduce infection risk is social distancing in indoor areas.

Marques (52) introduced a solution to CO_2_ real-time monitoring using an IoT system and called it the iAirCO_2_ platform. There are some sophisticated technological features of the iAirCO_2_ as it consists of Web as well as smartphone software for data consulting in addition to a hardware mock-up for the collection of latent data. Ultimately, the objective is for doctors to be able to access these data in the future to help them make a medical diagnosis. Compared to other systems, the iAirCO_2_ relies on open-source technology and offers a comprehensive wireless system with benefits such as scalability, flexibility, affordable pricing, and a simple installation. These findings demonstrate that the production of a system can serve as a credible indoor air quality assessment and facilitates the prediction of technical adjustments that lead to a better living environment.

Salman et al. (71) described the design and implementation of wireless sensor units to examine indoor air quality for real-time data capture. This is accomplished using infrared sensors that detect humidity, CO_2_, and temperature as well as low-power wireless networking and geographical prediction using geostatistical approaches. The platform contains an MBED LPC 1768 board that collects the sensor unit data and delivers it to a central base station through a ZigBee module where it is analyzed and stored.

Gilio et al. (27) conducted experiments, split into two evaluation stages, concerning CO_2_ levels real-time monitoring during the reopening of schools in the Apulia Region of southern Italy. The first evaluation was the preliminary procedure where classroom activities in nine schools (11 classrooms) were assessed. A detailed air ventilation operating protocol was also implemented to determine whether there would be an improvement. Even though the windows and doors were opened for air circulation during the first assessment stage, six classrooms (54%) reached CO_2_ concentration levels of more than 1,000 ppm. It means all the classrooms exceeded the prescribed CO_2_ levels of 700 ppm. The second evaluation stage sought to improve the conditions from the first phase by applying a detailed air ventilation operating protocol. NDIR sensors were deployed to simultaneously visualize real-time CO_2_ levels as part of the CO_2_ monitoring process.

Ng et al. (53) tested NDIR CO_2_ gas sensing by applying pyroelectric detectors that are CMOS compatible and MEMS ScAlN-based. The researchers used an eight-inch wafer level technology and 12 percent Sc-doped AlN stored at 200°C to create their ScAlN pyroelectric detectors. Using a blackbody thermal emitter, a 10-centimeter (cm) long enclosed gas channel with inlet and outlet holes connected to some tubing, and carrying out tests utilizing two types of reference gases (synthetic air and N_2_), the experiment resulted in a voltage signal drop. The drop is believed to be caused by the CO_2_ gas absorption—specifically at the 4.26 μm wavelength—at CO_2_ gas concentrations that ranged from 25 parts per million (ppm) to 5,000 ppm. The findings suggest that affordable, monolithic, wafer-level NDIR gas sensors with a minimal footprint that is merged with CMOS circuits are possible to achieve by using pyroelectric detectors.

Chojer investigated the development of low-cost indoor air monitoring devices and found that most devices were lack of assessment. Using the standard factory calibration setting and ML models such as Multiple Linear Regression, Support Vector Regression, and Gradient Boosting Regression as sensor calibrations were needed for data reliability, especially measuring the response time that played an essential role in real-time monitoring (15, 22).

## 4. Discussion

The COVID-19 outbreak in the Hubei province, specifically Wuhan, China had been initially considered pneumonia back in December 2019. The quick-spreading nature of this coronavirus disease became apparent as global case numbers rose and nations reported cases of the SARS-CoV-2 virus in their respective countries. This demonstrates how infectious diseases are frequently caused by viruses. Characteristics of diseases include being highly pathogenic and contagious as well as being easily transmissible in congested or poorly ventilated indoor spaces, such as health facilities and public locations. People spend nearly three-quarters of their day indoors hence exposing them to a variety of external and internal air contaminants.

For indoor environments, the quality of the ventilation construction system affects airborne transmission which is a crucial route for the spread of contagious viruses. The CO_2_ concentration of indoor spaces possesses a significant impact on airborne transmission by way of indoor air and it can impact mental activity, increase reading errors, reduce cognitive and behavioral responses, increase end-tidal CO_2_ levels, decrease heart rate, spur breathing problems, and cause unconsciousness (38). As such, an increase in CO_2_ concentration signals inadequate ventilation and, if an infected person is nearby, would boost the risk of a COVID-19 infection (1, 8, 72). In addition, humidity level and temperature have been found to exacerbate the transmission of the virus. Most research found a correlation between seasonality and increased COVID-19 cases where the spread of SARS-CoV-2 in the winter is faster than in summer (25).

Learning activities in educational buildings, such as classrooms were primarily observed as part of the monitoring system (27, 28, 30, 31, 39, 62, 65). The prevention of SARS-CoV-2 transmission inside such infrastructures and the safe reopening of some public spaces and educational facilities were implemented through mechanical or non-mechanical means. The mechanical strategy adopts intelligent air ventilation made up of sensors and the sensor data will be transmitted to the cloud computing system thus easing users in terms of monitoring and controlling (28, 65, 66). Nowadays, CO_2_ monitors are available in the marketplace and moreover, the Federation of European Heating, Ventilation and Air Conditioning Associations (REHVA) recommends installing CO_2_ sensors to alert against poor ventilation in indoor areas (27). Eleven of the fifty four conducted studies examined the use of sensors (31, 35, 36, 39, 43, 44, 49, 53, 56, 62, 63, 65, 66), and indicated that the NDIR sensor is the simplest and most common technology (8, 15). The mechanism of the NDIR sensor is to evaluate the value of light that is absorbed across wavelengths. Because CO_2_ absorbs light at wavelengths, other gases present provide minimal interference although temperature and humidity react with readout. Other than the NDIR sensor, the microcontroller ESP8266 can also support the creation of low-cost indoor air monitoring (31, 50). Powering the ESP8266 with a wireless module allows for the supervision of ambient temperature in real time. The results from the monitoring system are stored and projected on a screen that users can view every 30 min. Ensuring data reliability by calibrating the devices of the low-cost indoor air monitoring before they were used.

Constructing a building with a low-cost physical barrier of at least 60 cm in height above the desk surface and arranging the distance of personnel working areas (within 4 m of the outlet) (6) are some preventive measures to maintain a safe and healthy environment for open offices. Initiatives including lockdown restrictions, using facemasks, hand washing, practicing physical distancing of more than 2 m, and using disinfectants on surfaces in crowded places are supplementary, but also effective means.

## 5. Conclusion

By observing the indoor air quality of public places like health facilities, education facilities, offices, and other buildings, decision-makers can move forward with shaping and enforcing policies as well as programs to prevent the spread of COVID-19. Enhancing building ventilation systems and using low-cost assembly sensors are some of the ways to improve indoor air quality. The NDIR sensor and ESP8266 microcontroller are used to monitor CO_2_ which has a significant part in airborne transmission. Intelligent indoor monitoring mitigates the SARS-CoV-2 spread and the risks of other common symptoms such as coughs, headaches, eye irritation, dizziness, and fatigue. Innovative technologies may help alleviate the ongoing COVID-19 pandemic and help prevent similar global crises in the future. From an engineering perspective, sensors equipped with a cloud computing architecture can be used to build hygienic ventilation systems with low energy consumption. Other uncomplicated ways include wearing a face mask and maintaining a physical distance. Although there have been many contributions regarding this multidisciplinary subject, most are still in the early stages of development and require further refinement, providing an abundance of opportunities for researchers.

## Data availability statement

The raw data supporting the conclusions of this article will be made available by the authors, without undue reservation.

## Author contributions

Conceptualization: C-TY and P-YL. Methodology: RW, P-YL, and EK. Software: RW and C-TY. Validation: C-TY, T-KY, and P-YL. Formal analysis: C-TY. Investigation: P-YL. Resources: RW, EK, and C-TY. Data curation and visualization: RW. Writing: RW, C-TY, and P-YL. Supervision: C-YH, P-YL, and C-TY. All authors have read and agreed to the published version of the manuscript.
